# Tuberculous Aortic Aneurysm - A Review

**DOI:** 10.21470/1678-9741-2020-0611

**Published:** 2022

**Authors:** Abdulmajeed Altoijry

**Affiliations:** 1 Department of Surgery, Division of Vascular Surgery, King Saud University, College of Medicine, Riyadh, Saudi Arabia.

**Keywords:** Aortic Aneurysm, Blood Vessel Prosthesis, Tuberculosis, Early Diagnosis, Endovascular Procedures, Treatment Outcome

## Abstract

**Introduction:**

Tuberculous aortic aneurysm (TBAA) is an exceedingly rare but severe manifestation of tuberculosis, with a high risk of sudden rupture of the aorta in absence of medical or surgical intervention. This review aimed to provide a detailed understanding of TBAA, including its associated complications, affected population, treatment measures, and outcomes.

**Methods:**

Case studies and relevant research articles were analyzed to understand the recent advances in medical scientific knowledge on TBAA. Recent clinical case reports on TBAA were searched from the year 2010 to 2020.

**Results:**

Case reports indicated a higher prevalence of TBAA in the male population. The most affected age group was 15 to 79 years. The most common treatment for TBAA included surgery followed by antituberculous medication. The case reports discussed in this review reflected open surgery, endovascular repair, coil embolization, laparotomy, aortic valve and root replacement as some of the surgical procedures used depending on the complication and type of aneurysm. The treatment outcome was considered effective in most cases.

**Conclusion:**

Postoperative chemotherapy and medications reduce the risk of severity. Early diagnosis of TBAA is imperative, followed by surgical resection and postoperative antituberculous medication with careful follow-up to prevent relapse.

**Table:** Abbreviations, Acronyms & Symbols

AAA	= Abdominal aortic aneurysm
AFB	= Acid-fast bacilli
ATT	= Antituberculosis therapy
BCG	= Bacillus Calmette-Guérin
CT	= Computed tomography
DTA	= Descending thoracic aorta
FDA	= Food and Drug Administration
RIPE	= Rifampicin, isoniazid, pyrazinamide, and ethambutol
TAA	= Thoracic aortic aneurysm
TAAA	= Thoracoabdominal aortic aneurysm
TBAA	= Tuberculous aortic aneurysm
TB	= Tuberculosis
TEVAR	= Thoracic endovascular aortic repair

## INTRODUCTION

Aortic aneurysm is a chronic degenerative disease affected by the weakening of the aortic wall resulting in “ballooning” of an artery exceeding 1.5 times its normal diameter. Aortic aneurysms are 3-4 times more prevalent in men than in women^[1-3]^. They are generally silent and asymptomatic until aortic dissection or rupture occurs^[4]^, Depending on the spatial distribution of dilation, aortic aneurysm can be classified into three categories: thoracic aortic aneurysm (TAA), abdominal aortic aneurysm (AAA), and thoracoabdominal aortic aneurysm (TAAA).

Tuberculous aneurysm of the aorta (TBAA) is an exceedingly rare but severe manifestation of tuberculosis^[5,6]^. The first case of tuberculous involvement of the aorta (aortitis) was reported by Weigert in 1882, and no patient was known to have survived due to TBAA until the combined technologies of modern imaging capabilities, antituberculous drugs, and vascular grafts became available. In 1952, Herndon et al.^[7]^ attempted the first surgical repair of a tuberculous aortic aneurysm, but the patient died six days after surgery^[5,7]^. In 1955, Rob and Eastcott reported the first successful reconstruction of the condition using an orlon cloth graft^[5]^. TBAA is a severe condition due to the high risk of sudden rupture. This condition arises because of transmural perforation caused by direct extension to the vessel^[8]^.

TBAA is an extension of the tuberculosis (TB) infection. It occurs in the walls of the small pulmonary and meningeal arteries from the neighboring or contiguous inflammatory foci. It often causes aneurysms in the tuberculous cavities and meninges. The progression of infection occurs from the lungs to various parts of the body^[9,10]^. Tuberculous involvement of the aortic wall may occur mostly by the direct extension from contagious lesions such as infected lymph nodes, empyema, pericarditis, vertebrae or paraspinal abscess, and hematogenous or lymphangitic dissemination^[11-13]^. Almost all segments of the aorta may be involved in the site of infection. The sites of involvement include ascending aorta, distal aortic arch, proximal descending thoracic aorta, distal descending thoracic aorta, and infrarenal abdominal aorta^[11-13]^. However, the areas in close proximity to the mediastinal and para-aortic lymph nodes are more commonly involved. Early diagnosis and appropriate surgical or endovascular repair are necessary for successful management of patients with these complicated aneurysms.

The aim of this review was to provide a detailed understanding of TBAA, including its associated complications, affected population, treatment measures, and outcomes. To accomplish this, all possible case reports from the last ten years were summarized to evaluate diagnostic complications. Recommended treatment from those reports can provide a clear understanding of their respective risk factors and clinical management.

## METHODS

All literature databases such as PubMed, Embase, Medline, and Google Scholar were searched to retrieve the relevant literature for this narrative review. The search terms used were “Tuberculous aortic aneurysm, Clinical management, Chemotherapy, Antituberculous therapy, Risk factors, and Open surgical repair”. The search terms were combined using Boolean operators “AND/OR”. Forty-six recent clinical case reports on TBAA were searched from the year 2010 to 2020. Cross-references in the retrieved papers were also referred, if considered relevant.

## RESULTS

### Clinical Features of Tuberculous Aneurysm

Tuberculous aortitis typically occurs at the distal aortic arch and the descending aorta, which are close to specific groups of mediastinal lymph nodes, and very rarely occurs in the ascending aorta^[14]^. Tubercle bacilli can enter the aortic wall in the following ways: i) being directly implanted on the inner surface of the vessel wall; ii) moving to the adventitia or media through the vasa vasorum; and iii) entering the vessel wall through the lymphatics from a contiguous focus, including lymph node or paraspinal abscess^[10,15]^. Clinical features of TBAA vary greatly, ranging from asymptomatic aneurysm with or without constitutional symptoms like pulsatile or palpable mass, chest pain, dysphagia, hoarseness, abdominal pain, back pain, frank rupture, bleeding, and shock. Due to the wide variations in symptoms, the investigation becomes a major problem. Tuberculous aortic aneurysms are diagnosed using clinical symptoms, chest radiography, computed tomography (CT) scans, cardiovascular magnetic resonance, and angiography^[12,13]^. Therefore, patients are usually diagnosed at high index of suspicion. An aneurysm should be suspected in patients with active tuberculosis if they suddenly deteriorate or if a mass lesion is present^[8]^. Moreover, in case of delay in diagnosis, patients may be treated with immunosuppressive therapy before the diagnosis of TB^[16]^. Indeed, the possibility of tuberculous aortitis should be considered in patients with aortitis with a history of pulmonary and/or extrapulmonary tuberculosis with chronic immunosuppression. In these cases, suspicious findings are cavitary lung lesion, pleural effusion, or lymphadenitis. TBAA becomes severe due to aortic rupture leading to a life-threatening condition. Hence, treatment as soon as possible is recommended^[16,17]^.

### Pathophysiology

Infection of the aorta by *Mycobacterium tuberculosis (M. tuberculosis)* usually appears as the direct extension from a contiguous infection (*i.e*., tuberculous lymphadenitis) that leads to the formation of an aortic pseudoaneurysm^[10]^. The possibility of an infectious cause must always be considered as a treatment strategy for infectious and non-infectious aortitis^[18,19]^. The active state of the aortic aneurysm is defined by current diagnostic and classification criteria. A case study by Delaval et al.^[6]^ found that, from 2003 to 2015, of 86 patients admitted with aortitis, about 3.5% of cases had TBAA. The diagnosis of TBAA was challenging in this study due to the median delay of 18 months between the first symptom and the start of antituberculosis therapy (ATT). Aortic aneurysm infection of *Mycobacterium bovis* is one of the rare complications. Currently published literature reported about 22 cases of bacillus Calmette-Guérin (BCG) spondylitis after intravesical therapy. Among them, only four cases showed a combination of mycotic aortic aneurysm and BCG spondylitis^[17,20-22]^.

Recent reports from India suggested the possibility of additional pathologies including irregularities of the aortic lumen, stenotic lesions, and occlusion in young patients with TB elsewhere in the body, which are unrecognized in Western experience. The probable reasons for these lesions were hypersensitive reaction to the tuberculous antigens^[15]^.

### Risk Factors

Travel, migration, multidrug-resistant *M. tuberculosis* strains, marginalized populations (*e.g.*, the homeless or addicts), and immunosuppression, particularly acquired immunodeficiency syndrome, are important factors that contribute to the spread of tuberculosis causing aortic aneurysms^[23]^. Because of these factors, cardiovascular surgeons may be more frequently confronted with this pathology. Infants and young children (especially those under 2 years of age) are at greatest risk of developing severe, disseminated disease associated with high morbidity and mortality and they get infected usually by adults^[24]^.

### Gender and Age as Major Risk Factors for TBAA

**Fig. 1:**
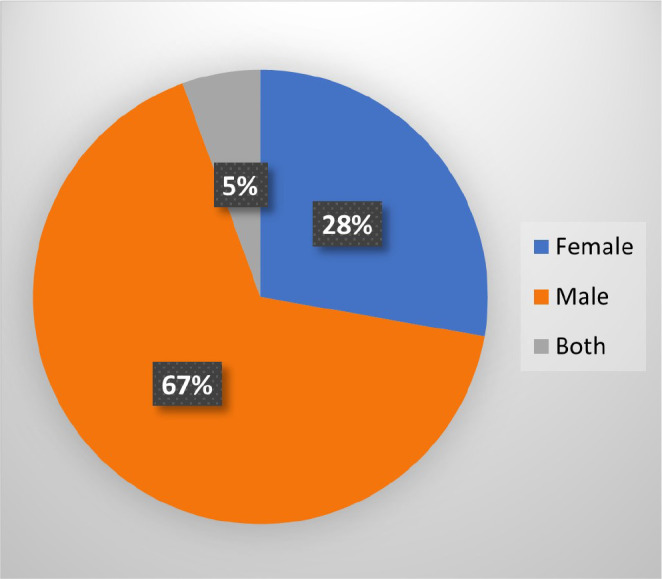
Gender as a risk factor for TBAA observed in the case reports from the last ten years.

Approximately 18 case studies were reported under TBAA in the last ten years (2010 to 2020). Out of these case studies, 12 studies reported about male, five about female, and one study reported about both male and female (Table 1). Data from all these studies were analyzed and it was found that about 67% of the case studies included males, 28% included females, and 5% included both males and females (Figure 1). In most of these studies, the patients belonged to the age group between 61 and 80 years. Hence, it seems that age and gender are the two most important factors affecting the prevalence of TBAA.

*TBAA=tuberculous aortic aneurysm.*

**Table 1:** Case reports of tuberculous aortic aneurysm.

Author & year	Study	Age (years)/gender (M/F)	Initial complications	Location (artery)	Surgical procedure	Follow-up (months)	ATT (months)		Outcome	Comment
							Pre-operative	Post-operative		
Clough et al., 2010^[31]^	Tuberculous mycotic aneurysm	49	Breathlessness, weight loss, and fever, back pain after 5 months of RIPE treatment	Supraceliac (originating from the celiac artery), infradiaphragmatic saccular, false aortic aneurysm	Endovascular repair using custom-made endovascular graft	48	5	3	Survived	Endovascular repair of a tuberculous mycotic aortic aneurysm using a custom-made stent graft may be both feasible and durable
Costiniuk et al., 2010^[32]^	Ruptured AAA and SFAA secondary to *M. bovis*, occurring 2 years after receiving BCG therapy	75/M	Abdominal pain and back pain without fever	AAA and pseudoaneurysm of the SFA	Emergency laparotomy with Dacron graft implantation 8 days after the vein graft interposition to bypass the left SFA	12	NR	12	Survived	Clinicians should be aware of the possible extravesical complications of BCG therapy
			History of hypertension, dyslipidemia, smoking, diabetes, and PMR							
Benjelloun et al., 2012^[28]^	Treatment of multiple TAA of tuberculous origin	16/F	Thoracic pain with a history of ruptured AA aneurysm	TAA	EVAR using Multilayer stent	6, 12, 18	No	No (aspirin and clopidogrel for 1 month)	Survived	EVAR with an uncovered stent is preferred to open surgery, preventing high morbidity and mortality
Pierret et al., 2012^[33]^	Multiple tuberculous aortic aneurysms of the thoracic and abdominal aorta	19/F	Fever, thrombocytopenic purport, gum bleeding, microcytic anemia, lymphocytosis, elevated CRP, and elevated fibrinogen	Dilation of AA, posterior thoracic and celiac aorta	Surgical resection with cryopreserved aortic allograft patch	1, 3, 6, 9	1.1	9	Survived	Early if not systematic detection of aortic tuberculous lesions in tuberculous patients plays a crucial role in the effective treatment of TAA
Kuhan et al., 2013^[35]^	Tuberculous abdominal aortic aneurysm on a patient with renal transplant	61/M	Lower back pain, malaise, night sweats, weight loss, end-stage renal disease, diabetes	AA, IA	EVAR	6	MIPE × 1 MAPE × 18	Yes, 18	Survived	EVAR is best for immunosuppressed patients with transplantation with TB mycotic aneurysms
Marjanovic et al., 2013^[34]^	Mycotic aneurysm of the thoracic aorta	63/M	Back pain, hemoptysis, temperature 38 °C, WBC count 12,000/L, and CRP value 150 mg/L	Mycotic aneurysm of DTA	EVAR	6, 12	No	4	Survived	Endoluminal stent graft is a better alternative for DTA treatment. Long-term postoperative ATT and regular follow-up are important
Holmes et al., 2014^[17]^	Mycotic aortic aneurysm due to intravesical BCG immunotherapy	64/M	Progressive back pain, smoking, high-grade bladder cancer	Proximal aortic aneurysm from the proximal abdominal aorta	Open surgical debridement. Partial left heart bypass and distal aortic perfusion		RIPE initiated held 72h prior to surgery	RIE × 3 RME × 1 RM × 6	Survived	Surgical evaluation and ATT (2 × RIE followed by IR × 7) is essential for mycotic aortic aneurysm
Kim et al., 2014^[36]^	Multiple tuberculous TAA	47/M	Persistent fever, left buttock abscess, left inguinal lymphadenitis	IAA, RCIA, DTA	EVAR, transcatheter embolization	14	1	Yes, 12	Survived	EVAR with antimycobacterial therapy is a treatment option for multiple tuberculous aneurysms
Pathirana et al., 2015^[37]^	Ascending aortic aneurysm with severe aortic regurgitation caused by *M. tuberculosis*	40/F	Exertional breathlessness (NYHA II), weight loss	TA (ascending), AR	Aortic valve and root replacement	10	RIPE × 10	Yes, 10	Survived	Clinical and radiological diagnostic criteria for tuberculous aortitis need to be optimized in the absence of apparent etiology
Velayudhan et al., 2016^[38]^	Multiple tuberculous mycotic aneurysms of the aorta extending from the distal aortic arch to the aortic bifurcation	18/M	Pulmonary TB, abdominal pain	TAA	Open surgical repair	NR	Yes	ATT × 6	Survived	Open graft replacement is an appropriate treatment for TB-related thoracoabdominal aneurysm
Dilangalen, 2016^[39]^	Multiple saccular aneurysms caused by *M. tuberculosis*	58/M	Severe abdominal pain	ScA, AA	Total arch replacement	NR	No	Yes, NR	Survived	Surgery and long-term ATT are the best option for tuberculous aortitis
Higashi Y 2018^[20]^	AAA caused by *M. bovis* after treatment with intravesicular BCG therapy for bladder carcinoma	69/M	Acute onset of intermittent stabbing pain in the right lower abdominal quadrant.	IAA	Surgical resection and aortic reconstruction	1, 6, 9	Yes	9	Survived	Cryopreserved aortic allograft for in-line reconstruction provides technical simplicity and long-term patency
			Fever, chills, and night sweats for 3 months							
Pluemvitayaporn et al., 2018^[40]^	Mycotic abdominal aortic aneurysm and lumbar tuberculous spondylitis with cauda equina syndrome	79/M	Severe back pain, low-grade fever, malaise, weight loss	AA (infrarenal)	Radical debridement by left transpsoas approach, then endovascular stent graft, finally posterior decompressive laminectomy	12	NR	ATT × 12	Survived	Successful management involves accurate diagnosis and prompt treatment
D’Cruz R 2019^[47]^	Multiple synchronous tuberculous aneurysms	35/F	Painless right supraclavicular lump, unintentional weight loss	TA, CA, LR & SA, RI	TEVAR, ascending aortic replacement with aortic valve suspension, coil embolization	12	Yes (NR)	6 months	Survived	Multidisciplinary management of synchronous TA is essential for the best clinical outcome
Li et al., 2019^[25]^	Ruptured thoracic aortic pseudoaneurysm secondary to Pott’s disease during spine surgery	57/F	Primary pulmonary TB, breathlessness, chest pain, weight loss, fever	TA	Posterior vertebra stabilization surgery (for Pott’s disease), endovascular treatment due to rupture, partial corpectomy	24	NR	Yes, 12	Survived	Repair or stabilization of TAA is necessary prior to spine surgery
Savlania et al., 2019^[41]^	Primary AEF due to tubercular aortitis	75/M	Black, tarry stool and hematemesis	IAA	Open surgical repair of an aneurysm with extra‑anatomical right axillounifemoral bypass	1	NR	RIPE, NR	Survived	Patients with AEF due to tuberculosis can be saved with early surgery
Zhao et al., 2019^[42]^	Mycotic aortic aneurysms with miliary TB	73/M	Pain in the back and right-side of the chest, dry cough, inability to walk. Based on pulmonary TB (under treatment)	TA, AA, RIA	EVAR	Patient died due to pulmonary infection	RIPE × 1	NR	Died	ATT therapy is inadequate, microcore stent graft is a possible option to improve hemodynamics
Mimbimi et al., 2020^[43]^	Dissecting aneurysm of ascending aorta secondary to Takayasu’s arteritis with concomitant tuberculosis	15/M	Growing right cervical mass, all arterial pulsations, vascular murmur in carotid and subclavian arteries on both sides	Aortic arch wall thickening, saccular dissecting aneurysm in the ascending aorta, several fusiform aneurysms and stenosis of all supra-aortic arteries	Ascending aorta and total aortic arch replacement	6 months	3 months	NR	Survived	There are very few reports on Takayasu’s arteritis with concomitant tuberculosis leading to aortic dissection. The patient was successfully managed in this case

A=amikacin; AA=abdominal aorta; AEF=aortoenteric fistula; AR=aortic root; ATT=antituberculous therapy; BCG=bacillus Calmette-Guérin; CA=coronary arteries; CRP=C-reactive protein; DTA=descending thoracic aorta; EVAR=endovascular stent graft repair; IA=iliac artery; IAA=infrarenal abdominal aorta; LR & SA=left renal and splenic arteries; M=moxifloxacin; NR=not reported; PMR=polymyalgia rheumatica; RCIA=right common iliac artery; RI=right internal iliac; RIA=right iliac artery; RIPE=rifampin, isoniazid, pyrazinamide, and ethambutol; SA=subclavian artery; ScA=subclavian artery; SFA=superficial femoral artery; SFAA=superficial femoral artery aneurysm; TA=thoracic aorta; TAA=thoracoabdominal aorta; WBC=white blood cells

### Clinical Management of Tuberculous Aneurysm

The accepted treatment of TBAA is a combination of antimycobacterial therapy and surgery. Neither medical treatment nor surgery is curative, if used alone. Availability of combined technologies of modern imaging, anti-TB therapy, and vascular grafts defines the rate of cure and durability^[5]^. Early recognition of aneurysms and prompt surgery by excision or grafting is found to be a prominent solution. The types of surgeries are debridement of the infected field with *in situ* prostheses, prosthetic grafting, extra-anatomic bypass, insertion of an aortic conduit, patch closure and direct closure^[6,9,25]^.

These surgeries appeared to offer outstanding results. However, surgery is associated with high rates of postoperative complications, such as surgical wound infection, nosocomial bacteremia, early graft infection, bleeding, cerebrovascular events, and respiratory problems^[11-13]^.

ATT is associated with steroids in case of inflammatory stenotic lesions. Surgery is not warranted in all cases, but it is suggested in case of worsening of the stenotic lesions followed by anti-inflammatory drugs^[26]^. Histopathological study and culture of the tissue from the aneurysm wall should be a routine procedure for the correct drug choice in these complicated patients^[11-13]^.

In the case report of a 54-year-old woman with miliary tuberculosis, a mycotic aneurysm of the descending aorta was incidentally discovered and successfully treated with antimycobacterial therapy and endovascular stent-graft placement with a complete resolution of the condition within one year^[27]^. Recently, endovascular treatment has emerged as an alternative to surgery for these aneurysms^[28]^. In 2005, Food and Drug Administration (FDA) approved thoracic endovascular aortic repair (TEVAR) as the primary treatment strategy for degenerative thoracic aortic aneurysms^[29,30]^. Type of surgery, drug medication and post-symptoms of some of the recent studies are presented in Table 2.

**Table 2:** Recent trends in surgery and drug management in TBAA.

Author	Surgery	Drug/medication	Symptoms
Li et al.^[25]^	Yes	Yes	Breathlessness, chest pain, weight loss, and fever
	Surgery consisting of posterior spine stabilization, anterior excision of the infected field, and aortic reconstruction. Endovascular stent grafting provides the best results immediately	1-year AT chemotherapy	
Han et al.^[27]^	Yes	Yes	Chest and back discomfort, painless right supraclavicular lump and unintentional weight loss
	Ascending aortic replacement with aortic valve suspension and coil embolization of right iliac artery pseudoaneurysm	ATT	
Velayudhan et al.^[38]^	Yes	Yes	Abdominal pain
	Open surgery	Isoniazid and rifampicin	
Dilangalen^[39]^	Yes	Yes	Severe abdominal pain
	Arch replacement surgery	ATT	
Pluemvitayaporn^[40]^	Yes	Yes	Severe back pain, low-grade fever, malaise, and weight loss
	Radical debridement via left transpsoas approach	AT chemotherapy for 12 months	
Savlania et al.^[41]^	Yes	Yes	Upper gastrointestinal bleeding, abdominal pain, and pulsating abdominal mass
		ATT	
Zhao et al.^[42]^	No	Yes	Pain in the back and on the right side of the chest associated with dry cough, presented with inability to walk for 1 month
	Endovascular repair with microcore stent graft	AT regimen (pyrazinamide, isoniazid, rifampicin, and ethambutol)	
Mimbimi et al.^[43]^	Yes	Yes	Growing right cervical mass, arterial pulsations, vascular murmur in carotid and subclavian arteries on both sides
		ATT, high-dose corticosteroids, antiplatelet therapy, betablocker administration during the initial active phase	
AT=antituberculous; ATT=antituberculous therapy.			

## DISCUSSION

### Different Cases Studies on TBAA

In 2010, Clough et al.^[31]^ presented a case report of a patient with initial complaints of breathlessness, weight loss, and fever, and was diagnosed with acid-fast bacilli (AFB) infection. After five months of rifampicin, isoniazid, pyrazinamide, and ethambutol (RIPE) medication, angiography suggested supraceliac, infradiaphragmatic saccular, false aortic aneurysm, which was repaired by endovascular repair using custom-made endovascular stent graft. The patient had no postoperative complications and was discharged after 3 days.

Costiniuk et al.^[32]^ (2010) presented a case of tumor resection and intravesical BCG immunotherapy for bladder. Emergent laparotomy was performed, and Dacron graft was implanted with subsequent antimycobacterial therapy (isoniazid, rifampicin, and ethambutol). The patient developed complications after 1^st^ and 4^th^ month due to hematomata with likely granulation tissue formation and a giant cell reaction to the foreign material but did not have AFB infection. The patient became stable after 12 months.

Pierret et al.^[33]^ (2011) reported a case of a female patient with history of fever, thrombocytopenic purport, and gum bleeding. CT scan of the chest and abdomen revealed celiomesenteric lymphadenopathy and saccular dilations of abdominal aorta, while magnetic resonance angiography confirmed the presence of four saccular dilations of the posterior thoracic and celiac aorta. Subsequently, a percutaneous lymphadenopathy biopsy demonstrated granulomas with caseous necrosis consistent with tuberculosis. After five weeks of ATT therapy, surgical resection with cryopreserved aortic allograft patch for the reconstruction was performed. The antituberculous regimen was continued over nine months with follow-up. The patient recovered well, with no sign of illness.

Benjelloun et al.^[28]^ (2012) reported a case of a female patient with a history of ruptured abdominal aortic aneurysm of tuberculous origin. Endovascular surgery was suggested, and three uncovered multilayer stents were implanted to cover the entire aneurysmal segment of the thoracoabdominal aorta above the renal arteries. Postoperative medications were aspirin and clopidogrel for one month. Follow-up after 18 months showed regression of certain aneurysms and disappearance of others over the course.

In 2013, Marjanovic et al.^[34]^ reported the case of two patients with aortic aneurysm, one of them with tuberculous origin. Urgent endovascular repairs of descending thoracic aorta (DTA) were successfully performed with stent graft. After confirmation of mycobacterial infection, ATT therapy was advised to the patient for four months. Six-month follow-up revealed no endoleak, and complete aneurysm thrombosis with regression of aneurysm diameter with no signs of infection after 1 year.

Kidney transplant in an immunocompromised patient was reported by Kuhan et al.^[35]^ (2013). Bronchoalveolar lavage was positive for AFB and initially treated with antituberculous drugs, such as moxifloxacin, isoniazid, pyrazinamide, and ethambutol. The patient was cured from endovascular aneurysm repair and later discharged with an 18-month course of anti-TB medication.

Kim et al.^[36]^ (2014) reported a case of multiple tuberculous aneurysms of the thoracic and abdominal aorta subjected to endovascular stent graft repair. The patient then continued antimycobacterial treatment for 1 year and had no fever two months after the procedure. Fourteen months after the procedure, the patient was stable without any recurrence.

Pathirana et al.^[37]^ (2015) reported a case of tuberculous aortitis. Clinical, imaging and histological findings confirmed ascending aortic aneurysm due to tuberculous aortitis. Aortic valve and root replacement were suggested with warfarin therapy along with ATT. The patient showed good clinical recovery on follow-up with functioning aortic prosthesis. Velayudhan et al.^[38]^ (2016) reported a case in which the patient with TBAA recovered well after open surgical repair. Dilangalen et al.^[39]^ (2017) reported a case of infectious aortitis with complaint of abdominal pain. The patient underwent total arch replacement, started with a fixed dose of ATT and fully recovered. Endovascular stent graft implantation proved to be an ultimate option for TBAA treatment in a 79-year-old male from Thailand^[25,40]^. The patient underwent posterior decompressive laminectomy and showed a gradual improvement in motor power. Finally, he was on continuous treatment with antituberculous chemotherapy for one year and was completely recovered^[40]^.

In the case study presented by Li et al.^[25]^ (2019), a patient undergoing pulmonary TB treatment was diagnosed with thoracic aortic pseudoaneurysm. Here, posterior spine stabilization was followed by the placement of an endovascular stent graft due to the unexpected rupture of the pseudoaneurysm. Afterwards, infected tissues were debrided, and then spinal fusion was performed. Finally, the patient was advised with antituberculous chemotherapy for one year. After 24-month follow-up, the patient was completely cured and showed no signs of recurrent infection.

Savlania et al.^[41]^ (2019) reported a case study on a saccular aneurysm arising from the anterior wall of the infrarenal aorta near the posterior wall of the duodenum. Open surgical repair of aneurysm with extra-anatomical right axillounifemoral bypass followed by exploratory laparotomy was performed and the patient was cured with some additional antitubercular drug.

Recently, a case by Zhao et al.^[42]^ (2019) reported a male patient with chronic obstructive pulmonary disease who was diagnosed with multiple aneurysms. Endovascular repair using a microcore stent graft was performed. However, the patient died due to a pulmonary infection acquired while recovering at a local hospital. Mimbimi et al.^[43]^ (2020) reported a case of aortic arch wall thickening, a saccular dissecting aneurysm in the ascending aorta, several fusiform aneurysms and stenosis of all supra-aortic arteries. Examination of the cervical mass revealed *M. tuberculosis* infection and subsequently advised with ATT. After three months, the patient reported acute chest pain and underwent ascending aortic arch replacement. Postoperative care was uncomplicated, and the patient was discharged on the 10^th^ day after surgery. No recurrence or evolution was seen after 6 months of follow-up.

### Prevalence, Recovery and Mortality

The predisposition to tuberculosis-related aortic aneurysm in the population residing in industrial areas is expected to be more prevalent^[44]^ However, studies on the epidemiology of tuberculosis-related aortic aneurysm worldwide are lacking, making it difficult to predict the risk factor for this disease. The age group of patients suffering from TBAA ranges from 18 to 79 years, with an average age of 59 years. Therefore, it is presumed that TBAA is more common in the older male population.

Medical therapy is suggested with a high dose of corticosteroids and immunosuppressive agents in case of steroid resistance. Prophylactic antibiotics, antituberculous drugs and antiplatelet therapy are the most common medications^[35,41]^.

Combined surgical and medical management of TBAA has accounted for the best outcomes. In some patients whose conditions are not suitable for open surgery, such as age, weakness or severe organ failure, stent graft combined with antituberculous drugs can be considered as the therapeutic regimen^[45]^. The recovery rate is high with ATT and surgery, showing a low mortality rate. Continuous follow-up is required for successful recovery of the patients after both surgery and ATT for at least 6-24 months.

The aneurysm could have been sterilized if the pre-existing lesion is taken in the aortic wall of vasa vasorum infected by *M. tuberculosis*^[46,47]^.

## CONCLUSION

Overall, although TBAA is a rare life-threatening condition, patients can be saved if early surgery is undertaken. It is more applicable in endemic countries having a maximum global burden of tuberculosis. Early clinical suspicion and diagnosis of the condition using chest radiography and CT scan or magnetic resonance imaging, followed by surgical resection and use of anti-TB medication, as well as careful postoperative follow-up to prevent relapse, are crucial to manage this fatal condition. More population-based studies are recommended to properly understand the risk factors and mortality rate for the effective management of this disease.

**Table:** Authors' roles & responsibilities

AA	Substantial contributions to the conception or design of the work; or the acquisition, analysis, or interpretation of data for the work; drafting the work or revising it critically for important intellectual content; agreement to be accountable for all aspects of the work in ensuring that questions related to the accuracy or integrity of any part of the work are appropriately investigated and resolved; final approval of the version to be published
